# Combination of HLA-DQ2/-DQ8 Haplotypes and a Single MSH5 Gene Variant in a Polish Population of Patients with Type 1 Diabetes as a First Line Screening for Celiac Disease?

**DOI:** 10.3390/jcm11082223

**Published:** 2022-04-15

**Authors:** Marta Wysocka-Mincewicz, Artur Groszek, Filip Ambrozkiewicz, Agnieszka Paziewska, Michalina Dąbrowska, Anna Rybak, Ewa Konopka, Agnieszka Ochocińska, Natalia Żeber-Lubecka, Jakub Karczmarski, Joanna B. Bierła, Ilona Trojanowska, Agnieszka Rogowska, Jerzy Ostrowski, Bożena Cukrowska

**Affiliations:** 1 Department of Endocrinology and Diabetology of the Children’s Memorial Health Institute, Aleja Dzieci Polskich 20, 04-730 Warsaw, Poland; m.wysocka@ipczd.pl (M.W.-M.); a.groszek@ipczd.pl (A.G.); 2 Department of Genetics, Maria Sklodowska-Curie National Research Institute of Oncology, Roentgena 5, 02-781 Warsaw, Poland; filip.ambrozkiewicz@lfp.cuni.cz (F.A.); michalina.dabrowska@pib-nio.pl (M.D.); natalia.zeber-lubecka@cmkp.edu.pl (N.Ż.-L.); j.karczmarski@cmkp.edu.pl (J.K.); jostrow@warman.com.pl (J.O.); 3 Laboratory of Translational Cancer Genomics, Biomedical Center, Faculty of Medicine in Pilsen, Charles University, Alej Svobody 1665/76, 323 00 Pilsen, Czech Republic; 4 Department of Gastroenterology, Hepatology and Clinical Oncology, Centre of Postgraduate Medical Education, 01-813 Warsaw, Poland; agnieszka.paziewska@cmkp.edu.pl (A.P.); arogowska@cmkp.edu.pl (A.R.); 5 Department of Neuroendocrinology, Centre of Postgraduate Medical Education, 01-813 Warsaw, Poland; 6 Faculty of Medical and Health Sciences, Institute of Health Sciences, Siedlce University of Natural Sciences and Humanities, 08-110 Siedlce, Poland; 7 Department of Gastroenterology, Great Ormond Street Hospital NHS Trust, Great Ormond Street, London WC1N 3JH, UK; anna.rybak@gosh.nhs.uk; 8 Department of Pathomorphology of the Children’s Memorial Health Institute, Aleja Dzieci Polskich 20, 04-730 Warsaw, Poland; e.konopka@ipczd.pl (E.K.); j.bierla@ipczd.pl (J.B.B.); i.trojanowska@ipczd.pl (I.T.); 9 Department of Biochemistry, Radioimmunology and Experimental Medicine of the Children’s Memorial Health Institute, Aleja Dzieci Polskich 20, 04-730 Warsaw, Poland; a.ochocinska@ipczd.pl; 10 Department of Oncological Gastroenterology, Maria Skłodowska-Curie National Research Institute of Oncology, 02-781 Warsaw, Poland

**Keywords:** type 1 diabetes, celiac disease, HLA-DQ2 haplotype, HLA-DQ8 haplotype, MSH5 gene, genetic screening

## Abstract

Patients with type 1 diabetes (T1D) are at increased risk for developing celiac disease (CD). The aim of the study was to assess the usefulness of celiac-specific human leukocyte antigen (HLA) haplotype and the rs3130484 variant of MSH5 gene, a previously described non-HLA variant associated with CD in the Polish population as a first-line screening for CD in T1D pediatric patients. Serological CD screening performed in the T1D group (*n* = 248) and healthy controls (*n* = 551) allowed for CD recognition in 20 patients (8.1%) with T1D (T1D + CD group). HLA-DQ2, HLA-DQ8 and the rs3130484 variant were genotyped with TaqMan SNP Genotyping Assays. The T1D + CD group presented a higher, but not statistically significant, frequency of HLA-DQ2 in comparison with T1D subjects. Combining the rs3130484 with HLA-DQ2/HLA-DQ8 typing significantly increased the sensitivity of HLA testing from 32.7% to 68.7%, and the accuracy of estimating CD prediction from 51.7% to 86.4% but decreased the specificity from 100% to 78.2%. The receiver operating characteristic curve analysis confirmed the best discrimination for the combination of both genetic tests with an area under curve reaching 0.735 (95% CI: 0.700–0.7690) in comparison with 0.664 (95% CI: 0.632–0.696) for HLA typing alone. Results show the low utility of HLA-DQ2/HLA-DQ8 typing for CD screening in T1D pediatric patients. Combination of the rs3130484 variant of the MSH5 gene and HLA testing increases both the sensitivity and the predictive value of the test accuracy, but still, the obtained values are not satisfactory for recommending such testing as the first-line screening for CD in T1D patients.

## 1. Introduction

Celiac disease (CD) is a common autoimmune disease with a prevalence of about 1% in Europe which is triggered by exposure to dietary gluten in genetically predisposed individuals [1,2]. Early CD diagnosis and treatment, based on a gluten-free diet, prevent many complications including the development of other autoimmune diseases, osteoporosis, or malignancy (especially T-cell lymphoma) [3,4,5]. However, due to the atypical (non-classical) or even asymptomatic clinical presentation, CD is often undiagnosed, or CD diagnosis is delayed [6]. Among groups at high risk of CD are first-degree relatives of patients with CD and patients suffering from type 1 diabetes (T1D), autoimmune thyroiditis, autoimmune hepatitis, selective immunoglobulin (Ig) A deficiency, and some genetic syndromes (e.g., Down’s syndrome, Turner syndrome) [7].

Almost all patients with CD carry DQ2 heterodimers of human leukocyte antigens (HLA-DQ2) consisting of alpha and beta subunits encoded by DQA1*05 or DQA1*02 and DQB1*02 alleles (haplotype HLA-DQ2.5 or HLA-DQ2.2, respectively) and/or HLA-DQ8 encoded by DQB1*03:02, generally in combination with the DQA1*03 variant [8]. In 2012, the European Society for Paediatric Gastroenterology, Hepatology and Nutrition (ESPGHAN) presented new guidelines for CD screening in at-risk groups, which suggested that genetic testing of the HLA-DQ2/-DQ8 haplotype should precede a serological testing that would only be performed in patients with a celiac-specific HLA genotype [7]. However, such an approach is controversial and not a cost-effective screening, especially in the group of T1D patients, because of the frequent association of T1D with risk haplotypes for CD [9,10,11]. On the other hand, although over 95% of CD patients have the HLA-DQ2 and/or HLA-DQ8 haplotype, it is not sufficient for CD development, as approximately 60% of CD genetic risk depends on genes not related to the HLA system [12]. The international Genome Wide Association Study (GWAS) identified 57 single nucleotide polymorphisms (SNPs) associated with CD [13], among which the strongest statistical significance was found for the SNP located within the LPP gene [14]. The meta-analysis covering six populations (Great Britain, Italy, the Netherlands, Poland, Spain, and India) presented that among European countries, only in the Polish population was the association of SNP within the LPP gene with CD not confirmed, similarly to the Hindu population. These results showed that CD-associated SNPs showed population specificity. In 2015, we identified a single SNP variant, rs3130484, located within the MSH5 gene, which was associated with CD in the Polish population, and presented that combination of HLA-tagging SNPs and the rs3130484 SNP improved the sensitivity of predicting CD risk in the group of first relatives of CD patients [15].

The aim of this study was to evaluate the frequency of celiac-specific haplotypes and the rs3130484 variant of the MSH5 gene in T1D patients to assess the usefulness of these genetic tests for CD screening.

## 2. Materials and Methods

### 2.1. Subjects

All enrolled patients and control subjects were from a Polish population (Caucasians). The study cohort consisted of 248 T1D pediatric patients under 18 years old (median age 8; 137 females and 111 males), 287 pediatric patients with recognized CD under 18 years (median age 4; 185 females and 102 males), and 551 healthy individuals recruited from Polish blood donors for other GWAS projects (median age 28 years; 403 females and 148 males). The control group was older than the CD and T1D groups, however, according to recently published analyses showing that study cohorts combining the younger cases with the older controls may significantly improve the discovery power of GWAS [16], the age of the groups in our study should not affect the obtained results.

T1D was diagnosed at the Department of Endocrinology and Diabetology of the Children’s Memorial Health Institute in Warsaw (CMHI) according to the International Society for Pediatric and Adolescent Diabetes (ISPAD) diagnostic criteria (hyperglycemia >200 mg/dL, and the presence of at least one diabetes-specific antibody: against glutamic acid decarboxylase, anti-tyrosine phosphatase, and/or anti-islet antibodies determined with the use radio ligand or enzyme-linked immunosorbent assays (Medipan GmbH, Blankenfelde-Mahlow, Germany)). A group of children with only CD was diagnosed at the Department of Gastroenterology, Hepatology and Nutrition of CMHI according to ESPGHAN guidelines [7,17]. CD was recognized in subjects with positive celiac-specific antibodies and histological changes described at least as Marsh 2. Some patients (36 out of 286, 12.6%) with a high concentration (>100 AU/mL, e.g., 10 times higher than the upper normal limit) of anti-tissue transglutaminase antibodies of immunoglobulin A (tTG-IgA) (Thermo Scientific Phadia GmbH, Freiburg, Germany), positive anti-endomysial antibodies (Euroimmune, Medizinische Labordiagnostika AG, Lubeck, Germany), and haplotype HLA-DQ2 and/or HLA-DQ8 (Thermo Fisher ScientificLife, MA, USA) were diagnosed with CD without intestinal biopsy [15]. Serological screening for CD was performed in the T1D group and controls and allowed for CD recognition in 20 out of 248 children (8.1%) with T1D (group T1D + CD). Nobody in the healthy control group had positive celiac antibodies. The patients’ characteristics are presented in Table 1. The age of children with T1D was significantly higher than children with CD (*p* < 0.01) and diabetic children with CD (*p* < 0.01).

### 2.2. Serological CD Screening

Serological CD screening tests were performed in T1D patients and healthy controls. The screening involved the measurement of tTG-IgA and antibodies against deamidated gliadin peptides of the IgG class (DPG-IgG). The tests were performed with the use of a Phadia 100 analyzer and EliA commercial kits (Thermo Scientific Phadia GmbH, Freiburg, Germany). When tTG-IgA were negative (the level < 10AU/mL) and DPG-IgG were positive (the level ≥ 10 AU/mL), the concentration of total IgA to confirm or exclude IgA deficiency was measured. In the case of positive tTG-IgA (regardless of DPG-IgG level) or in the case of IgA deficiency and positive DPG-IgG, a biopsy of the small intestine was always suggested. Histological examination of duodenal specimens confirmed CD-specific changes with an increased level of intraepithelial lymphocytes, crypt hyperplasia, and villous atrophy in all antibody-positive T1D patients [18].

### 2.3. DNA Isolation

Genomic DNA was extracted from whole blood treated with EDTA using a QIAamp DNA Blood Mini Kit (Qiagen, Hilden, Germany). DNA sample concentrations were assessed with a NanoDrop ND-1000 spectrophotometer (NanoDrop Technologies, Wilmington, DE, USA).

### 2.4. SNP Genotyping

Individual genotyping of patients and healthy controls was performed with the use TaqMan SNP Genotyping Assays (Thermo Fisher Scientific, Waltham, MA, USA for HLA typing: rs2187668 (HLA-DQ2.5), rs7454108 (HLA-DQ8), rs7775228, rs2395182 (HLA DQ2.2), and rs3130484 for the MSH5 gene [15]. SNP characteristics are presented in Table 2. DNA was genotyped with TaqMan™ Universal Master Mix II, no UNG (Thermo Fisher Scientific, Waltham, MA, USA) and a 7900HT Real-Time PCR system (Life Technologies, Waltham, MA, USA). The following amplification parameters were used: 10 min at 95 °C, followed by 40 cycles of 10 s at 95 °C and 1 min at 60 °C.

### 2.5. Statistics

Haplotype statistical analysis was carried out in R package (version 4.1.1). A Fisher test was applied to establish statistical significance (*p* value < 0.05). The odds ratios (ORs) and 95% confidence intervals (CIs) were estimated by normal approximation with a small sample adjustment (EpiTools R package). The Benjamini–Hochberg algorithm was used to correct *p*-values for multiple hypothesis testing. Specificity, sensitivity, positive predictive value (PPV), negative predictive value (NPV), and Receiver Operating Characteristic (ROC) curves for a CD occurrence model with the calculation of the area under the ROC curve (AUC) were evaluated with the use of Statistica v.10.0 software (StatSoft, Inc., Tulsa, OC, USA).

## 3. Results

### 3.1. HLA-DQ Haplotype Occurrence in T1D Patients

All groups were tested for DQ alleles sensitive to CD. HLA-DQ2 and/or HLA-DQ8 haplotypes were present in 84.21% of T1D patients and in all CD subjects and were significantly higher in both of these groups compared with controls (Table 3). The HLA-DQ8 haplotype was significantly more frequent (*p* = 5.98 × 10^−20^) in the T1D than in the CD group, and occurred in 56.14% and 17.01% of patients, respectively. In contrast, DQ2 haplotypes, both HLA-DQ2.2 and HLA-DQ2.5, occurred in significantly lower percentages in diabetic patients compared with the celiac group. Diabetic patients with CD presented a significantly higher frequency of HLA-DQ2, especially HLA-DQ2.5, in comparison with T1D subjects without CD. However, after *p* correction for multiple hypotheses, *p*-values between those groups did not reach statistical significance. Although all patients with T1D + CD carried HLA-DQ2 and/or HLA-DQ8, these results were not significant in comparison with T1D children without CD.

### 3.2. rs3130484 Typing for CD Screening in T1D Patients

We presented earlier that a single rs3130484 variant of the *MSH5* gene was significantly associated with CD [15], and this result was confirmed in the present study (Table 4). Almost 70% of celiac patients carried rs3130484, whereas this SNP was present only in 17.79% of controls (*p*= 3.87 × 10^−49^, OR = 10.4). The *MSH5* SNP occurred with significantly higher frequency in T1D patients than in controls (*p* = 3.01 × 10^−7^; OR = 2.54), but the presence of this polymorphism was significantly lower in comparison with the CD group: 35.5% and 69.3%, respectively (*p* = 4.15 × 10^−14^, OR = 4.1). Diabetic patients with CD presented with a similar frequency of the rs3130484 SNP as CD patients without statistical significance: 60% and 69.34%, respectively. Although the rs3130484 variant occurred more often in T1D + CD patients in comparison with T1D patients without CD, this result did not achieve statistical significance. Addition of the rs3130484 SNP to celiac-specific HLA typing significantly increased the screening power for CD in the group of T1D, but after *p*-value correction for multiple hypothesis testing using the Benjamini–Hochberg algorithm *p*-values for all combinations (MSH + HLA-DQ2, MSH + HLA-DQ2.5, and MSH + HLAQD2/HLA-DQ8), this did not achieve statistical significance (*p* = 0.06).

### 3.3. Diagnostic Value of CD-Specific Haplotypes and the rs3130484 Variant of the MSH5 Gene

**Figure 1:**
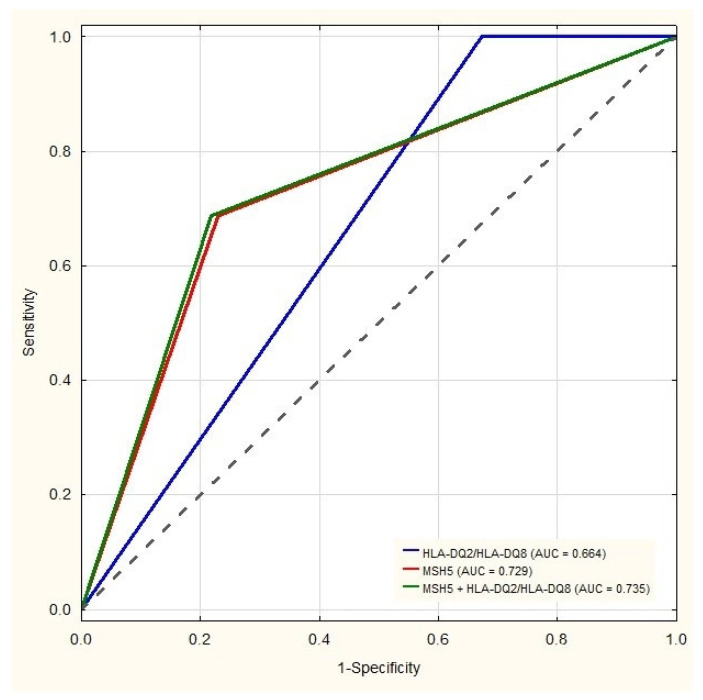
Receiver Operational Characteristics (ROC) analysis presenting specificity and sensitivity of CD haplotypes (HLA-DQ2/HLA-DQ8), the rs3130484 variant of the MSH5 gene (MSH5), and a combination of MSH5 and HLA-DQ2/HLA-DQ8 (MSH5 + HLA-DQ2/HLA-DQ8) in the detection of CD in patients with T1D. Area under curve (AUC) for HLA-DQ2/HLA-DQ8 (DQ2/DQ8) = 0.664, 95% CI: 0.632–0.696; for MSH5 = 0.729, 95% CI: 0.694–0.763 for MSH5 + HLA DQ2/HLA DQ8 (MSH5 + DQ2/DQ8 = 0.735, 95% CI: 0.700–0.769).

To assess the diagnostic value of the combination of HLA and non-HLA rs3130484 variant within the MSH5 gene, sensitivity, specificity, ACC, PPV, and NPV were analyzed (Table 5). The sensitivity and specificity of the routinely assessed HLA-DQ2/HLA-DQ8 were 100% and 32.7%, respectively. The ACC estimating CD prediction for the T1D group was 51.7%. The combined analysis of the MSH5 SNP variant and HLA-DQ2/HLA-DQ8 haplotypes increased the ACC to 86.4% while changing the sensitivity and specificity to 68.7 and 78.2%, respectively. The ROC analysis (Figure 1) confirmed that adding the MSH5 variant to the HLA-DQ2/HLA-DQ8 testing improved the AUC. Combination of HLA-DQ2/HLA-DQ8 and the rs3130484 variant of the MSH5 gene showed the best discrimination with the AUC reaching 0.735 (95% CI: 0.700–0.769). An improvement of CD risk prediction by using the proposed model was statistically significant (*p* < 0.01).

## 4. Discussion

The coincidence of CD with T1D is widely known and oscillates in the range from 1.6% and 12.3% [19,20,21]. The meta-analysis based on 26,605 patients with T1D enrolled from different countries found a prevalence of biopsy-confirmed CD of 6.0% [22]. Despite the fact of CD and T1D co-existence, challenges remain in establishing CD diagnosis in T1D because of the absence of symptoms suggestive of CD in the majority of diabetic patients [23,24]. The long-term effects of unrecognized CD in T1D patients are not well established, but accelerated diabetes-related complications are suggested [25,26,27]. Undiagnosed CD in T1D children can lead to poorer metabolic control and increased risk of hypoglycemia and retinopathy in consequence. T1D patients are therefore at risk of CD developing, and CD screening of them regardless of symptoms is recommended [7,28]. All experts agree that one-time serological screening tests for the presence of specific celiac antibodies are not sufficient. The ISPAD recommends performing serological screening at the onset of T1D, and then every 1–2 years for a period of 5 years [28]. The American Diabetes Association (ADA) recommends serological testing soon after diagnosis of T1D, and then after 2 and 5 years, or more frequently, if symptoms suggestive of CD appear [29]. As serological tests need to be repeated periodically and the risk of CD developing in case of negative HLA-DQ2/-DQ8 is very low, ESPGHAN experts suggest genetic HLA screening as a first-line screening test for CD diagnosis in at-risk groups including T1D [7]. However, in contrast to other risk groups, for example, first-degree relatives, such an approach in diabetic patients appears to be suboptimal due to the high frequency of the HLA-DQ2/HLA-DQ8 haplotype occurring in T1D [9,10,11]. In the current study, we found celiac-specific HLA haplotypes in 84.6% of T1D patients, and this result is compatible with the results of other researchers [30,31,32,33]. We report 100% sensitivity of HLA testing for CD screening, but low specificity, achieving 32.7%. Although all patients with CD, including those with T1D and CD, have carried HLA-DQ2 and/or HLA-DQ8 haplotypes (NPV = 100%), a high frequency of these haplotypes have been observed in a healthy control population that has resulted in low values of PPV and ACC: 36.9% and 51.7%, respectively. However, the addition of the rs3130484 variant localized within the MSH5 gene to HLA-DQ2/HLA-DQ8 testing increased the diagnostic significance of genetic screening in T1D patients. A combination of the HLA-tagging SNPs and the MSH5 SNP increased sensitivity to 68.7% and decreased specificity to 78.2%, but significantly increased the ACU in ROC analysis and predictive value of the test accuracy (ACC) to 86.4%. Similar results were obtained in our earlier analysis performed on the first-degree relatives of CD patients, that is, individuals who, like T1D patients, are at high risk of CD developing [15]. Thus, the current study confirmed that improvement of CD risk prediction sensitivity could be achieved by including the rs3130484 SNP of the MSH5 gene to the celiac-specific HLA haplotypes in genetic testing. Romanos et al. also presented that combining HLA and non-HLA variants associated with CD improved the identification of potential CD patients in the at-risk groups [34]. However, unlike our study where we added a single SNP, they combined the HLA study with 57 non-HLA SNPs that were presented in GWAS as associated with CD [13].

The current study presents that the rs3130484 SNP could discriminate between T1D patients with high risk of CD. The diagnostic value of this single SNP testing is even higher than that for HLA testing: AUC for HLA-DQ2/HLA-DQ8 = 0.664 (95% CI: 0.632–0.696) and for the rs3130484 variant = 0.729 (95% CI: 0.694–0.763). Adding HLA genetic testing to the rs3130484 SNP only slightly increased the diagnostic value of such screening (AUC = 0.735, (95% CI: 0.700–0.769)).

The MSH5 gene is located on chromosome 6p21.3 and codes the protein product, playing a crucial role in mismatch repair and meiotic homologous recombination [35]. SNPs located within the MSH5 gene (but non-rs3130484) were reported to be associated with various human diseases including neoplasia, reproductive disorders, immune deficiencies (selective IgA deficiency, common variable immune deficiency), and autoimmune diseases, such as systemic lupus erythematosus, Kawasaki disease, and T1D [36]. Valdes and Thomson identified the rs707915 SNP of the MSH5 gene in a block of six other markers linked through linkage disequilibrium as the second strongest T1D susceptibility marker [37]. In the current study, we also observed a significantly higher frequency of the rs3130484 SNP in T1D patients (35.5%) than in controls (17.8%, OR = 2.54 (1.77–3.66), *p* = 3.01 × 10^−7^) that could suggest that the SNP located within the MSH5 gene might affect autoimmunity development both in CD and T1D.

In summary, our results have shown that the implementation of HLA genotyping as a first-line screening tool has to be reconsidered because it is not distinctive, and we have presented that the addition of a single rs3130484 SNP within the MSH5 gene into HLA tests increases the power of CD screening in T1D. We believe that a combination of celiac-specific HLA testing and the MSH5 variant may be used in clinical practice as supporting the selection of CD high-risk T1D patients not only in Polish but also in other populations. It should be emphasized that the addition of MSH5 genotyping into HLA analyses does not significantly increase the costs of genetic tests. Considering the costs of CD-specific serological tests which should be frequently performed in T1D patients, the screening algorithm, including combination of the MSH5 variant and specific celiac HLA SNPs, and then serological testing only the genetically selected risk group seems to be cost-effective. However, further studies confirming the results in other populations with a greater number of analyzed patients that could increase the sensitivity of genetic screening tests are necessary.

### Strengths and Limitations of the Study

This study for the first time presents that a single rs3130484 SNP localized within the MSH5 gene added into genetic HLA testing may improve CD risk prediction sensitivity in T1D patients. The main strength of the study is homogeneous, clinically well characterized patient groups with long-term follow-up for CD and T1D.

The study presents few limitations, the foremost being the low case number in the group of patients presenting with T1D and CD. However, it should be emphasized that this group was an effect of the screening program in the whole population of T1D patients in our center, which reflects the population prevalence in Polish children. Additionally, lack of a probe allowing us to distinguish between HLA-DQ4 and HLA-DQ2.2 haplotypes [38] may introduce bias in data analysis.

## 5. Conclusions

The results show the low utility of CD-specific HLA-DQ2/HLA-DQ8 testing as a first-line screening for CD in T1D pediatric patients. An improvement of CD risk prediction sensitivity could be achieved by adding a single rs3130484 SNP localized within the MSH5 gene into genetic HLA testing. Combination of the MSH5 SNP and HLA-DQ2/HLA-DQ-8 testing increases both the sensitivity and the predictive value of the test accuracy, however, the obtained values are still not satisfactory to serve as the first screening test in T1D patients.

## Figures and Tables

**Table 1:** Patients’ characteristics in studied groups.

	T1D	T1D + CD	CD	Healthy Controls
Total number	228	20	287	551
Median age (in years)	9	5 *	4 *	28
Age range (in years)	9/12–18	2–11	1–17	18–68
Female number (%)	126 (55.3%)	11 (55.0%)	185 (64.5%)	403 (73.1%)
Male number (%)	102 (47.3%)	9 (45.0%)	102 (35.5%)	148 (26.9%)

Serological CD screening was performed in all T1D patients. CD was recognized in 20 T1D children (8.1%). * The ages of T1D + CD patients and CD patients were significantly lower in comparison with T1D patients.

**Table 2:** Characteristics of SNPs used in the study.

SNP	Assay ID *	Haplotype	Region/Gene	Allele/MAF **	Genotype
rs7454108	c_298171179_10	DQ8	N/A	C = 0.1018	(T;T)
					(C;T)
					(C;C)
rs2187668	c_58662585_10	DQ2.5	HLA-DQA1: Intron Variant	T = 0.1194	(T;T)
					(C;T)
					(C;C)
rs2395182	c_11409965_10	DQ2.2	HLA-DRA: 500B Downstream Variant	G = 0.2075	(T;T)
					(G;T)
					(G;G)
rs7775228	c_29315313_10	DQ2.2	N/A	C = 0.1287	(T;T)
					(C;T)
					(C;C)
rs3130484	C__30535385_10	MSH5	MSH5: Intron Variant	C = 0.0990	(T;T)
					(C;T)
					(C;C)

* Assay ID = commercial (Thermo Fisher Scientific, Waltham, MA, USA) identifier of SNP Assay; ** MAF = Minor Allele Frequency across European Population, http://www.ncbi.nlm.nih.gov/snp (accessed on 10 October 2021); N/A = not available.

**Table 3:** The frequency of HLA-DQ2 and HLA-DQ8 haplotypes in patients with diabetes mellitus type 1 (T1D), T1D and celiac disease (T1D + CD), celiac disease (CD), and healthy controls.

Haplotype	T1D *n* = 228	T1D + CD *n* = 20	Statistics T1D vs. T1D + CD			CD *n* = 287	Statistics T1D vs. CD		Controls *n* = 551	Statistics T1D vs. Controls	
	*n* (%)	*n*(%)	*p*	OR (95% CI)	*p* #	*n* (%)	OR (95% CI)	*p* #	*n* (%)	OR (95% CI)	*p* #
HLA-DQ8	128 (56.1%)	13 (60%)	0.49	1.4 (0.5–4.5)	0.54	49 (17.1%)	6.2 (4.1–9.5)	5.98 × 10^−20^	98 (17.79%)	5.90 (4.15–8.44)	3.72 × 10^−24^
HLA-DQ2.5	99 (43.4%)	16 (80%)	0.002	5.2 (1.6–22.0)	0.04	234 (81.53%)	5.7 (3.8–8.7)	1.10 × 10^−18^	136 (24.68%)	2.34 (1.67–3.28)	5.05 × 10^−7^
HLA-DQ2.2	38 (16.7%)	2 (10%)	0.75	1.8 (0.4–16.6)	0.79	112 (39.0%)	3.2 (2.1–5.0)	3.05 × 10^−8^	142 (25.77%)	0.58 (0.38–0.87)	0.008
HLA-DQ2	128 (65.1%)	17 (85%)	0.02	4.4 (1.2–24.1)	0.06	276 (96.2%)	19.5 (10.0–41.7)	2.34 × 10^−28^	263 (47.73%)	1.40 (1.02–1.94)	0.04
HLA-DQ2.2/HLA-DQ8 *	156 (68.4%)	15 (70%)	1	1.1 (0.4–3.6)	1	161 (55.1%)	1.8 (182–2.6)	0.002	222 (40.29%)	3.23 (2.31–4.56)	137 × 10^−12^
HLA-DQ2.5/HLA-DQ8 *	173 (75.9%)	20 (100%)	0.009	6.2 (0.8–215.2)	0.05	248 (86.4%)	2.0 (1.2–3.2)	0.004	223 (40.47%)	473 (330–6.85)	2.28 × 10^−19^
HLA-DQ2/HLA-DQ8 *	192 (84.2%)	20 (100%)	0.09	3.7 (0.5–131.4)	0.14	287 (100%)	53.5 (6.7–1787.0)	6.31 × 10^−14^	332 (60.25%)	3.54 (2.36–5.42)	5.03 × 10^−11^

* The number of patients with both haplotypes is not equal to the sum of the number of patients with the individual haplotypes, due to the presence of two haplotypes in the same patient. # *p* values after the correction for multiple hypothesis testing by the Benjamini–Hochberg algorithm. As differences in statistical significance before and after the correction were found only for analyses of T1D versus T1D + CD, *p*-values before correction were shown only for both of these groups.

**Table 4:** The frequency of the rs3130484 variant of the MSH5 gene (MSH5) and HLA-DQ2/DQ8 haplotypes in patients with diabetes mellitus type 1 (T1D), T1D and celiac disease (T1D + CD), celiac disease (CD), and healthy controls.

Genotype	T1D *n* = 228	T1D + CD *n* = 20	Statistics T1D vs. T1D + CD			CD *n* = 287	Statistics T1D vs. CD		Controls *n* = 551	Statistics T1D vs. Controls		Statistics CD vs. Controls	
	*n* (%)	*n*(%)	*p*	OR (95%CI)	*p* #	*n* (%)	OR (95%CI)	*p* #	*n* (%)	OR (95%CI)	*p* #	OR (95%CI)	*p* #
MSH5	81 (35.5%)	12 (60%)	0.05	2.7 (1.0–8.0)	0.09	199 (69.3%)	4.1 (2.8–6.0)	4.15 × 10^−14^	98 (17.8%)	2.54 (1.8–3.7)	3.01 × 10^−7^	10.4 (7.4–14.8)	3.87 × 10^−49^
MSH5 + HLA-DQ8	113 (49.6%)	13 (65%)	0.39	1.7 (0.5–5.0)	0.47	256 (89.2%)	2.3 (1.4–4.0)	0.001	226 (41.0%)	17.08 (7.8–42.7)	4.11 × 10^−18^	7.3 (3.2–18.8)	7.22 × 10^−8^
MSH5 + HLA-DQ2.5	102 (44.7%)	16 (80%)	0.03	2.9 (1.0–8.5)	0.06	234 (81.5%)	4.3 (3.0–6.4)	3.99 × 10^−15^	150 (27.2%)	2.89 (2.0–4.2)	2.08 × 10^−^^8^	12.5 (8.8–18.0)	2.03 × 10^−54^
MSH5 + HLA-DQ2.2	131 (57.5%)	17 (85%)	0.45	1.9 (0.0–17.3)	0.52	276 (96.2%)	10.1 (4.2–29.3)	6.94 × 10^−11^	272 (49.4%)	1.04 (0.3–2.9)	1	10.5 (5.6–20.8)	1.28 × 10^−17^
MSH5 + HLA-DQ2	163 (71.5%)	19 (95%)	0.03	2.9 (1.0–8.5)	0.06	219 (76.3%)	4.3 (3.0–6.4)	3.99 × 10^−15^	188 (34.1%)	2.70 (1.9–3.9)	1.00 × 10^−^^7^	11.7 (8.3–16.7)	1.74 × 10^−52^
MSH5 + HLA-DQ2.2/ HLA-DQ8 *	175 (76.8%)	20 (100%)	0.27	1.8 (0.6–5.2)	0.35	248 (86.4%)	1.4 (0.9–2.1)	0.13	235 (42.65%)	7.08 (4.1–12.6)	5.81 × 10^−14^	9.8 (5.9–16.9)	9.59 × 10^−24^
MSH5 + HLA-DQ2.5/ HLA-DQ8 *	185 (81.1%)	19 (95%)	0.03	2.8 (1.0–8.3)	0.06	287 (100%)	4.3 (2.9–6.3)	1.10 × 10^−14^	235 (42.65%)	2.94 (2.0–4.3)	1.32 × 10^−^^8^	12.5 (8.8–18.0)	2.03 × 10^−54^
MSH + HLA-DQ2/ HLA-DQ8 *	194 (85.1%)	20 (100%)	0.03	2.8 (1.0–8.3)	0.06	287 (100%)	4.3 (2.9–6.3)	1.10 × 10^−14^	339 (61.52%)	2.68 (1.85–3.87)	1.07 × 10^−7^	11.4 (8.0–16.2)	7.39 × 10^−52^

* The number of patients with both haplotypes is not equal to the sum of the number of patients with the individual haplotypes due to the presence of two haplotypes in the same patient. The statistical analyses were performed with the use of Fisher exact test. # *p*-value after correction for multiple hypothesis testing using the Benjamini–Hochberg algorithm. As differences in statistical significance before and after the correction were found only for analyses of T1D versus T1D + CD, *p*-values before the correction were shown only for these groups.

**Table 5:** The sensitivity, specificity, accuracy (ACC), positive predictive value (PPV), and negative predictive value (NPV) of CD-specific haplotypes and the rs3130484 variant of the MSH5 gene (MSH5) in detecting CD in patients with T1D.

Haplotypes/MSH5	Sensitivity (%)	Specificity (%)	PPV (%)	NPV (%)	ACC (%)
HLA-DQ2	95.8	49.8	42.9	96.8	62.8
HLA-DQ2.2	77.2	76.9	56.8	89.5	77.0
HLA-DQ2.5	41.7	69.8	35.3	75.2	61.9
HLA-DQ8	20.2	71.0	21.5	69.3	56.6
HLA-DQ2.2/HLA DQ8	85.3	51.5	40.9	89.9	61.0
HLA-DQ2.5/HLA-DQ8	58.0	49.2	31.0	74.8	51.7
HLA-DQ2/HLA-DQ8	100	32.7	36.9	100	51.7
MSH5	68.7	77.0	74.7	54.1	86.2
MSH5 + HLA-DQ2.2	65.1	77.4	90.9	77.6	85.3
MSH5 + HLA-DQ2.5	21.8	79.2	29.3	72.0	63.0
MSH5 + HLA-DQ8	11.1	93.1	38.6	72.6	69.9
MSH5 + HLA-DQ2.2/HLA-DQ8	67.1	90.5	73.6	87.5	83.9
MSH5 + HLA-DQ2.5/HLA-DQ8	30.9	78.8	36.5	74.3	65.3
MSH5 + HLA-DQ2/HLA-DQ8	68.7	78.2	75.5	55.4	86.4

## Data Availability

The datasets generated and/or analyzed during the current study are not publicly available but are available from the corresponding author by reasonable request.
